# Anti-Allergic Activity of Fucoidan Can Be Enhanced by Coexistence with Quercetin

**DOI:** 10.3390/ijms232012163

**Published:** 2022-10-12

**Authors:** Masashi Mizuno, Asuka Fujioka, Shiho Bitani, Ken-ichiro Minato, Hiroyuki Sakakibara

**Affiliations:** 1 Graduate School of Agricultural Science, Kobe University, 1-1 Rokkodai-cho, Nada-ku, Kobe 657-8501, Japan; 2 Department of Applied Biological Chemistry, Graduate School of Agriculture, Meijo University, 1-50 Shiogamaguchi, Nagoya 468-8502, Japan; 3 Faculty of Agriculture, University of Miyazaki, Gakuen Kibana-dai Nishi, Miyazaki 889-2192, Japan

**Keywords:** anti-allergic activity, fucoidan, polyphenol, galectin-9, quercetin, kaempferol

## Abstract

In recent years, the incidence of type I hypersensitivity including hay fever has been increasing year by year in Japan. Our previous study using mice showed that only oral, but not intraperitoneal, administration of fucoidan extracted from seaweed (*Saccharina japonica*) suppressed type I hypersensitivity by secretion of galectin-9, which has a high affinity for IgE in the blood. However, the amount of seaweed required to achieve this activity is quite high (12 g dry weight per person per day). Therefore, the purpose of this study was to search for food ingredients in vegetables that enhance type I hypersensitivity suppression effect when consumed together with fucoidan. As a result, the enhanced effect was observed in extracts from Welsh onions and onions among vegetables. When we compared the polyphenols in the vegetables that showed activity with those that did not, flavonols such as quercetin and kaempferol were found as candidates. When quercetin or kaempferol (100 μg each) were orally administered at the same time, even at amounts where fucoidan alone showed no anti-allergic activity, anti-allergic effects were observed. More interestingly, when both flavonols were combined and administered simultaneously at half the amount of each of the above flavonols (50 μg), while the fucoidan amount remained the same, a similar effect was observed as when each flavonol (100 μg) was administered alone. The simultaneous intake of fucoidan and vegetables containing high contents of quercetin or kaempferol may reduce fucoidan intake while maintaining the allergy suppression effect, suggesting the importance of food combination.

## 1. Introduction

Fucoidan is a fucose-rich sulfated polysaccharide that is found in the cell wall of brown sea algae and some marine invertebrates [1]. It is mainly composed of fucose and sulfated fucose, and its core region comprises primarily a polymer of α-(1→3) linked fucoses with a sulfate group substituted in the C-4 position of some fucose residues [2]. Fucoidan has been found in various brown sea algae and sometimes, it contains other minor sugar residues, such as mannose, glucose, xylose, and glucuronic acid, depending on the species of brown algae [2]. Reportedly fucoidan exhibits various biological activities such as antioxidant [1], anti-inflammatory [3], anti-tumor [4], anti-coagulant [5,6], and anti-viral effects [7]. More importantly, it was shown that fucoidan could exert anti-allergic effects [8,9].

Our previous studies revealed that only oral, but not intraperitoneal, administration of F-fucoidan extracted from seaweed (*Saccharina japonica*) suppressed type I hypersensitivity by secretion of galectin-9 (Gal9), which has a high affinity for IgE [10]. IgE antibody production is the major event leading to immediate hypersensitivity reactions. Moreover, we reported that the level of IgE attached on mast cells was decreased when sensitized mast cells were incubated with Gal9. This result indicated that Gal9 removes IgE from mast cells, thereby resulting in a decrease in the amount of IgE attached to mast cells, and thus inhibiting mast cell activation and suppressing allergy development [11]. Thus, oral administration of fucoidan may be effective in preventing and improving allergies. However, based on the results of the mouse experiment, the estimated dosage for humans was calculated to be about 12 g of dry seaweed per day, which is quite difficult to consume in daily life. Recently, it has been reported that the combination of food factors can enhance their functional activity more than when consumed alone with each other. Chakrabarti and Ray [12] reported that the combination of luteolin and silibinin showed synergistic anti-tumor action. Combination of quercetin and luteolin was demonstrated to increase luteolin permeability to the basolateral compartment of the in vitro co-culture systems, resulting in the observed anti-inflammatory and anti-allergic activities [13]. It has also been reported that the combination of *Enterococcus faecalis* IC-1 and luteolin promoted dendric cells to secrete IFN-γ and inhibited Th0 differentiation into the Th2 subset through suppressing GATA-3 expression [14]. Thus, polyphenols seemed to be attractive compounds that have the potential to enhance the anti-allergic activity. In this study, we searched for vegetables that enhance antiallergic activity by simultaneous intake with fucoidan and aimed to identify polyphenols contained in them.

## 2. Results

### 2.1. Anti-Allergic Effect Using a Combination of Fucoidan and Vegetable Extract in Passive Cutaneous Anaphylaxis Reaction

**Figure 1:**
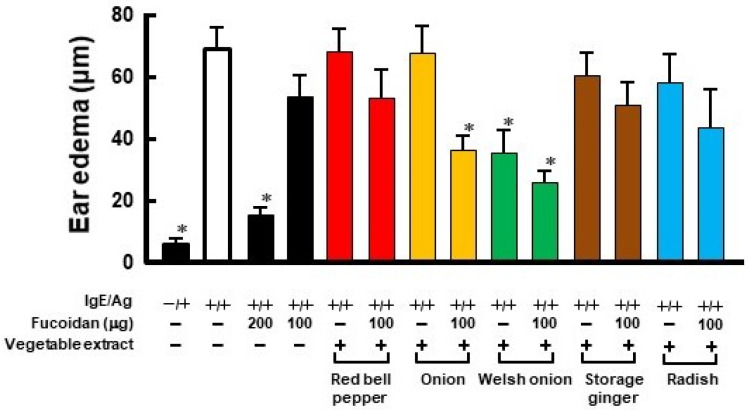
Anti-allergic activity of vegetable extracts in PCA reaction. Mice were orally administered test samples for 4 days and PCA reaction was conducted. Each vegetable extract dose was determined by converting it to correspond to the amount of extract extracted from 1 g of fresh vegetables. Ear edema was evaluated 2 h after antigen challenge. Values represent the means ± SE of 5 mice in each group. * *p* < 0.05, significant differences from the values of the group of IgE/Ag.

PCA reaction was developed as an animal model of mainly human type I allergic inflammation reaction [15]. As shown in Figure 1, oral administration of fucoidan (200 μg/day) suppressed ear edema but not 100 μg/day in PCA reaction. While onion extract itself did not show any activity, a combination of fucoidan in an inactive amount and onion extract, when administered orally, suppressed ear edema. Although Welsh onion alone possessed an inhibition of ear edema, its combination with fucoidan indicated a stronger inhibitory effect. However, each extract from red bell paper, storage ginger, and radish showed no activity when combined with fucoidan. Thus, it was clear that some vegetables enhanced the allergy-suppressing effect of fucoidan while others did not.

### 2.2. Polyphenol Contents in Various Vegetables

**Figure 2:**
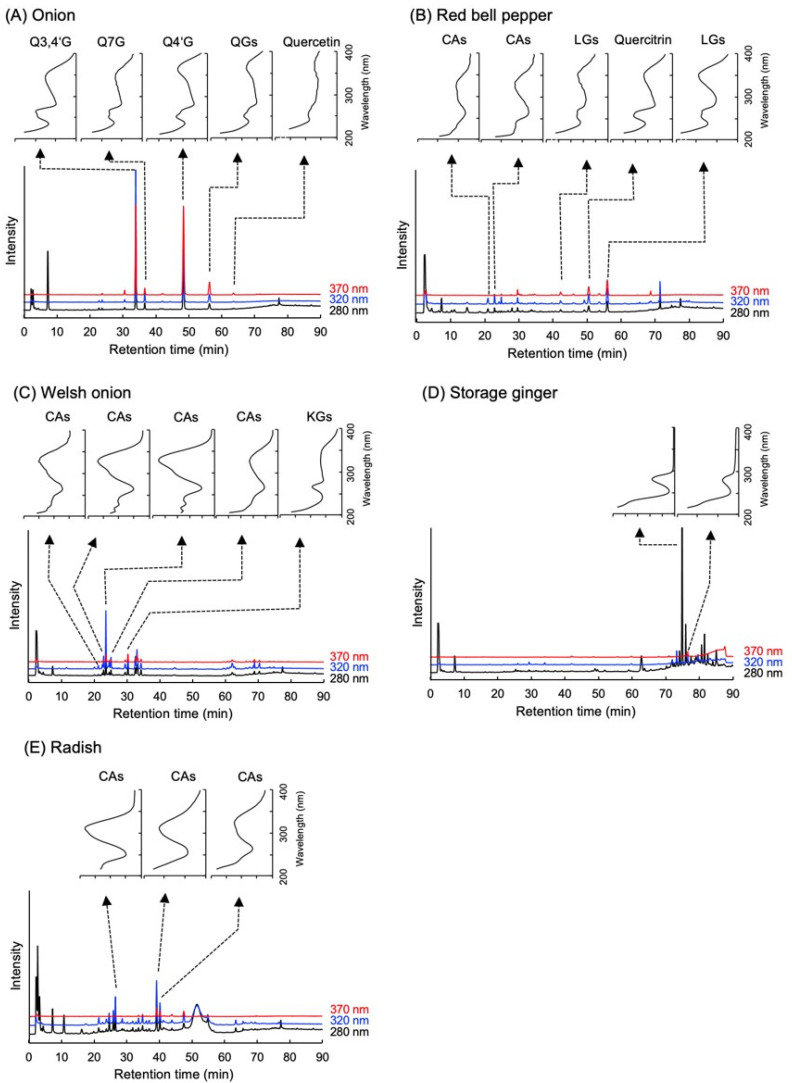
Typical HPLC chromatogram and UV-spectrum of (**A**) onion, (**B**) red bell pepper, (**C**) welsh onion, (**D**) storage ginger, (**E**) radish. Q3,4′G, quercetin 3,4’-diglucosides; Q7G, quercetin-7-glucoside; Q4′G, quercetin-4’-glucoside; QGs, quercetin glucosides; CAs, cinnamic acids; LGs, luteolin glucosides; KGs, kaempferol glucosides.

Polyphenol amounts were evaluated using the HPLC-DAD system. Using the present methods for polyphenol analysis, four major peaks were obtained in the onion extract (Figure 2A). Among these peaks, three peaks at retention time 33.8, 36.7, and 48.3 min coincided with quercetin-3,4′-diglucosides, quercetin-7-glucoside, and quercetin-4′-glucoside, respectively. On the other hand, the peak at 56.3 min did not coincide with the other quercetin glycosides in the library (quercetin-3-galactoside, quercetin-3-glucopyranoside, quercetin-3-rhamnoside, quercetin-3-rutinoside), but its photodiode array spectrum was very similar to that of quercetin aglycone. The onion powder was then hydrolyzed and analyzed again. Hydrolysis gave a single peak at a retention time of 63.6 min, which coincided with standard quercetin aglycone in retention time and spectra. This meant that all the four glycosides consisted of quercetin aglycon. We then calculated it as quercetin aglycone with the calibration curve of quercetin. The polyphenol amounts that existed in the other four vegetables were also calculated with similar methods, and their results were summarized in Table 1. Quercetin glycosides and kaempferol glycosides were abundant in onion and Welsh onion, respectively (Figure 2A,C), which enhanced the anti-allergic effect by co-existence with fucoidan. On the other hand, red bell pepper, which showed less effect, contained luteolin glycosides and trace amounts of quercetin glycosides (Figure 2B). Both storage ginger and radish (Figure 2D,E), which also showed less effect, were rich in cinnamic acids. These results implied that quercetin and kaempferol might be the active candidates that stimulated the anti-allergic effects with fucoidan.

### 2.3. Anti-Allergic Activity of Coexistence of Fucoidan and Quercetin or Kaempferol in PCA Reaction

**Figure 3:**
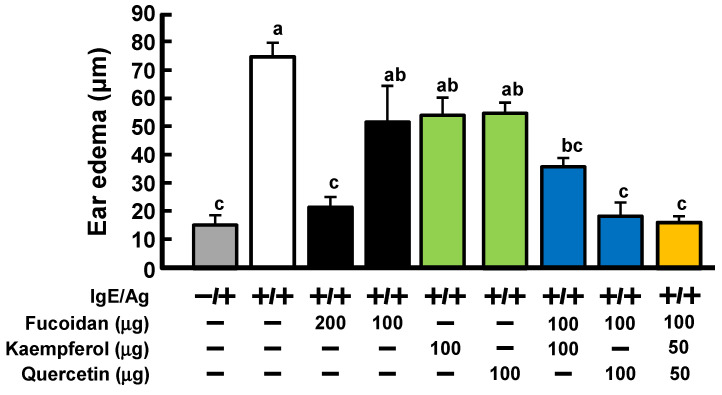
Anti-allergic activity of combination with quercetin or/and kaempferol in PCA reaction. Balb/c mice were divided into 9 groups and were orally administered fucoidan alone, kaempferol alone, quercetin alone, fucoidan + kaempferol, fucoidan + quercetin, and fucoidan + kaempferol + quercetin. After 4 days of administration, the PCA reaction was performed. Values represent the means ± SE of 5 mice in each group. Different letter (a, b, c) means the significant differences (*p* < 0.05; Tukey-Kramer test).

To confirm whether quercetin or kaempferol enhanced the anti-allergic activity of fucoidan, they were administered orally simultaneously at amounts that do not exhibit anti-allergic activity alone. As shown in Figure 3, 100 μg of either fucoidan, quercetin, or kaempferol alone did not show any activity when they were taken. However, simultaneous administration of 100 μg fucoidan and 100 μg of either quercetin or kaempferol significantly suppressed ear edema. In particular, the simultaneous administration of quercetin with fucoidan possessed a higher inhibitory effect than kaempferol. More interestingly, oral administration of 100 μg of fucoidan and 50 μg each of quercetin and kaempferol suppressed ear edema to almost the same levels as the control.

### 2.4. Involvement of Gal9 in Anti-Allergic Activity by Orally Simultaneous Administration of Fucoidan and Quercetin

**Figure 4:**
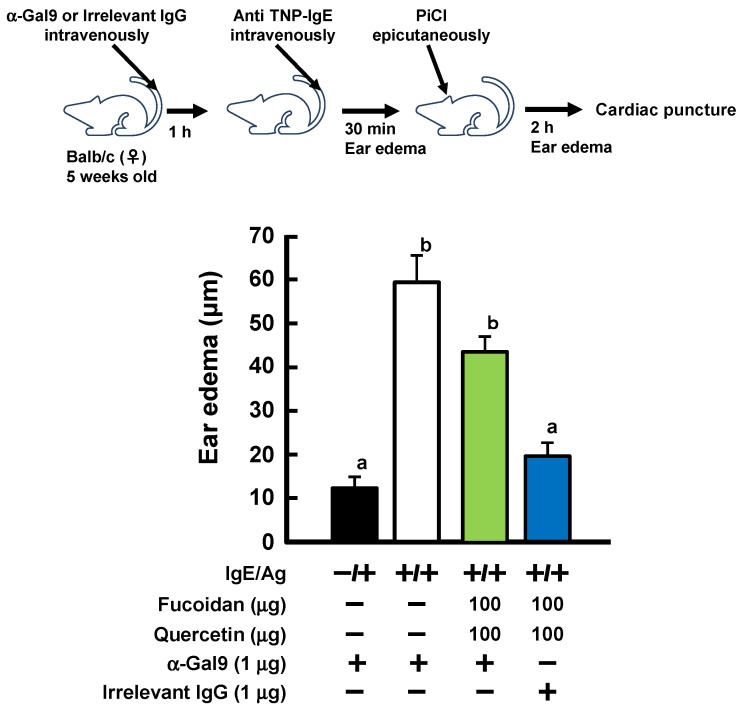
Involvement of galecti-9 in inhibitory effect of fucoidan and quercetin on PCA reaction. Mice were administered fucoidan and quercetin simultaneously for 4 days, and then intravenously injected with anti-gal9 antibody or isotype IgG 1 h before PCA reaction. Anti-TNP-IgE antibody was then intravenously injected and ear thickness was measured. Thirty minutes later, PiCl was applied to ear for challenge. Values represent the means ± SE of 5 mice in each group. Different superscript letter (a, b) means the significant differences (*p* < 0.05; Tukey-Kramer test).

To investigate the involvement of Gal9 in the development of the anti-allergic effect in the PCA reaction under the coexistence of fucoidan and quercetin, we tried to interfere with Gal9 action in the blood by tail vein injection of an anti-Gal9 antibody. As shown in Figure 4, ear edema, which was suppressed by oral administration of fucoidan and quercetin, were cancelled by injection of the neutralizing Gal9 antibody, but not isotype IgG antibody. These results suggested that Gal9 is involved in the suppression of allergy by simultaneous oral administration of fucoidan and quercetin.

### 2.5. Effect of Extract from Broccoli and Okra on Anti-Allergic Activity of Fucoidan in PCA Reaction

**Figure 5:**
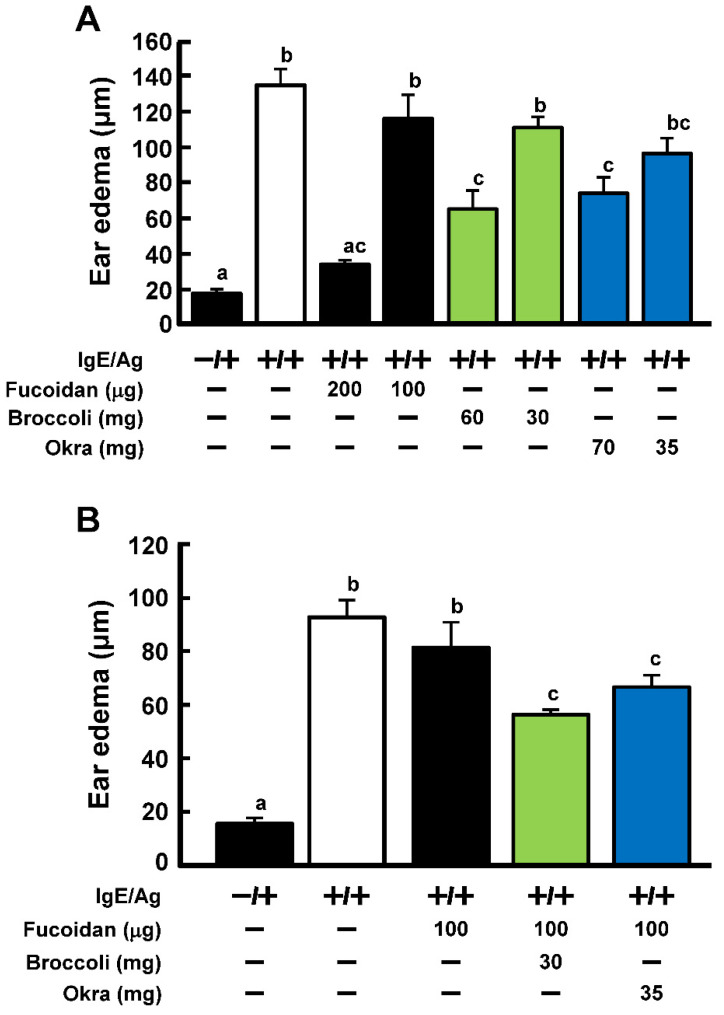
Anti-allergic activity of extracts from broccoli and okra in PCA reaction. (**A**) To determine the amount of each sample alone that did not possess an anti-allergic activity, mice were orally administered fucoidan, broccoli or okra extract for 4 days. (**B**) Mice were orally administered fucoidan and broccoli or okra extract for 4 days. After administration, PCA reactions were performed. Ear edema was evaluated 2 h after antigen challenge. Values represent the means ± SE of 5 mice in each group. Different superscript letter (a, b, c) means the significant differences (*p* < 0.05; Tukey-Kramer test).

We further explored the quercetin- and kaempferol-rich vegetables and fruits. Among 56 vegetables and fruits obtained in the local supermarket, okra (total quercetin glycosides = 12.5 μmol/g extracts) and broccoli (total kaempferol glycosides = 10.0 μmol/g extracts) were found to be the richest in quercetin and kaempferol, respectively. As shown in Figure 5A, the extract of broccoli and okra possessed anti-allergic activities at 60 and 70 mg, respectively. However, each sample did not at half the amounts. Fucoidan alone (200 μg) reduced ear edema significantly to almost half compared to the allergic induction group (IgE and antigen), but not in the 100 μg dose group. However, when oral administration of fucoidan (100 μg) was simultaneously administered with the extracts of broccoli or okra (30 mg and 35 mg, respectively), the ear edema was significantly suppressed to 69 and 81%, respectively, compared to fucoidan alone administration (Figure 5B). Thus, vegetables that contain high concentrations of quercetin and kaempferol were expected to synergistically possess the enhancement of the anti-allergic activity of fucoidan.

## 3. Discussion

We demonstrated that F-fucoidan, a polysaccharide in *Saccharina japonica*, ameliorates type I hypersensitivity by secreting Gal9 in blood by oral administration but not intraperitoneally [10]. Furthermore, it was confirmed that its oral administration eliminated IgE on the surface of mast cells and improved allergies even after allergen sensitization [11]. Fucoidan can be seen as an attractive compound that can help to prevent or improve allergies without affecting Th1/Th2 balance. However, in order to expect the benefits demonstrated by fucoidan, humans must consume a large amount of seaweed, as much as 12 g per day.

As a possible solution to this problem, we hypothesized that the intake of fucoidan could be reduced by combining food components. Recently, polyphenols are attractive phytochemicals exerting some physiological activities such as allergic inhibition. Singh et al. [16] have reviewed the effects of polyphenols on two critical reactions of allergic responses, sensitization to a given allergen and re-exposure to it. Quercetin lowered mast cell degranulation of bone-marrow mast cells (BMMCs) and human cultured mast cells, while no effect was observed for the glycosylated form of quercitrin [17,18]. Luteolin can inhibit mast cell degranulation as well as its structurally related polyphenol quercetin at lower concentrations [19]. Kaempferol suppressed β-hexosaminidase activity from RBL-2H3 cells when Caco-2 cells in co-culture system composed of Caco-2 cells in apical side and RBL-2H3 cells in basolateral side were stimulated with kaempferol, but its activity was lower than quercetin [20]. Cao et al. [21] reported that kaempferol is used as Parkinson disease protein 7 modulator for preventing mast cell-mediated allergic disorders through attenuating Lyn activation. While promising evidence has been found for single food factors, there have been reports that combination of food factors is also beneficial, including mixtures of vitamin D3, quercetin, and *Perilla frutescens*, and mixtures of vitamin D3 and *Lactobacillus reuteri* [22]. Similarly, we had confirmed that simultaneous intake of *Enterococcus faecalis* IC-1 and luteolin suppressed allergy in OVA-induced allergic mice [14].

As shown in Figure 1, it was found that vegetable extracts of onion and Welsh onion enhanced the allergic effect of fucoidan while red bell paper, storage ginger, and radish did not. This difference in the activity was presumably due to the kind or content of polyphenols contained in the vegetables. As a result of analyzing the amount of polyphenols in vegetable extracts by HPLC (Figure 2), it was confirmed that the vegetable extracts that showed allergy-suppressing effects contained high amounts of quercetin glycosides and kaempferol glycosides when administered orally at the same time as fucoidan at an amount that did not cause allergy suppression. In order to search for vegetables that contain higher amounts of quercetin and/or kaempferol, HPLC analysis was performed on 56 kinds of vegetables and fruits and each peak was assigned with a previously constructed polyphenol library [23]. The results indicated that broccoli and okra were identified as candidate vegetables, because they were the most quercetin- and kaempferol-rich vegetables among 56 vegetables and fruits, respectively. Furthermore, PCA reactions were performed on their extracts. Oral administration of 30 mg of broccoli and 35 mg of okra alone did not suppress ear edema. However, these two vegetable extracts significantly suppressed ear edema in the presence of fucoidan in the PCA reaction, even though they showed no activity when administered alone. Flavonoids including quercetin and kaempferol are widely distributed in the plant kingdom as mainly their glycoside forms, but their aglycone forms are little [23]. Drawing from fundamental knowledge, it is known that dietary flavonoids (glycoside forms) are poorly absorbed into the body in their intact forms. When these dietary flavonoids (glycoside forms) are consumed, they are enzymatically hydrolyzed to their aglycone forms by, for example, lactase-phlorizin hydrolase and cytosolic β-glucosidase in the intestinal tract [24], indicating that the physiological effects of flavonoids are mainly exerted by their aglycone forms, but not by glycoside forms. Therefore, we employed authentic quercetin and kaempferol aglycone in order to confirm that the anti-allergic activities of vegetable extracts depend on the amount of polyphenol contained in each vegetable. From the polyphenol library, it was estimated that 30 mg of broccoli and 35 mg of okra contained approximately 100 μg of the equivalent quercetin and kaempferol, respectively; therefore, quercetin or kaempferol was orally administered with fucoidan for the PCA reaction. Oral administration of each polyphenol showed significant anti-allergic activity equivalent to 200 μg fucoidan alone. It is interesting to note that oral administration of quercetin and kaempferol with fucoidan showed the same antiallergic effect at half the dose of the individual flavonoids when given alone with fucoidan. Further experiments will be needed to elucidate the cause of these findings. Furthermore, a study using an anti-Gal9 antibody in the tail vein injection revealed that the anti-allergic activity by oral administration of fucoidan together with flavanols is related to the production of Gal9 in the blood (Figure 4). Since the allergic suppressive effect of flavonoids is known to be due to Ca^2+^ influx and PKC inhibition on mast cells [17], it is further necessary to clarify the action of quercetin on mast cells in order to elucidate the mechanism of anti-allergic activity enhanced by a combination of fucoidan and quercetin.

## 4. Materials and Methods

### 4.1. Materials

Mouse anti-2,4,6-trinitrophenyl (TNP) monoclonal IgE was purchased from BD Biosciences (San Jose, CA, USA). 2,4,6-Trinitrochlorobenzene was purchased from Tokyo Chemical Industry (Tokyo, Japan). Sodium carboxymethyl cellulose (CMC) was purchased from Nacalai Tesque (Kyoto, Japan). An anti-mouse galectin-9 antibody and a normal goat IgG control were purchased from R&D System, Inc. (Minneapolis, MN, USA). Quercetin and kaempferol were purchased from Sigma-Aldrich (St. Louis, MO, USA) and Extrasynthese (Genay, France), respectively.

### 4.2. Mice

Female BALB/c mice (4 weeks old) were purchased from Japan SLC (Shizuoka, Japan). Mice were housed in an air-conditioned animal room at a temperature of 23 ± 2 °C and 55 ± 10% humidity under a 12-h dark/light cycle. Mice were acclimatized for almost 1 week, during which time food and water were provided ad libitum. This study was approved by the Institutional Animal Care and Use Committee and carried out according to the Kobe University Animal Experimentation Regulations (permission number: 29-02-02).

### 4.3. Passive Cutaneous Anaphylaxis Reaction

Mice were intravenously sensitized with anti-TNP IgE and then challenged by application of 1.6% 2,4,6-trinitrochlorobenzene in acetone:olive oil (1:1) as the antigen to the ear 30 min after sensitization according to our previous protocol [10]. Ear thickness was measured using a micrometer (Ozaki MFG., Tokyo, Japan) before and 2 h after antigen challenge, and the difference in ear thickness was defined as edema. Before the passive cutaneous anaphylaxis (PCA) reaction, mice were orally administered the test samples dissolved in distilled water or 0.5% CMC solution at appropriate concentrations every day for 4 days. To investigate the involvement of Gal9 production in the inhibitory effect of simultaneously oral administration of fucoidan and quercetin on PCA reaction, each mouse was injected intravenously with an anti-Gal9 antibody or irrelevant IgG 1 h before the injection of anti-TNP IgE.

### 4.4. Extraction of Polyphenol Fractions from Vegetables

Extraction and preparation of polyphenol fractions were performed according to previously used methods [23]. Briefly, domestic fresh vegetables including onion, red bell pepper, Welsh onion, radish, and storage ginger were obtained from local markets in Kobe City, Japan, during the summer season in 2019. After washing with tap water, the edible portions were pulverized with a mortar and pestle in liquid nitrogen, and then powders were lyophilized. The lyophilized powdery samples were extracted by shaking overnight at room temperature after adding (*w*/*v*) of 80% aqueous ethanol 20 times, and then the suspension was filtrated to remove insoluble matter. The filtrates were completely evaporated to remove solvent, and the residue was kept under –20 °C until the following experiments.

### 4.5. Analyses of Polyphenol Contents in Vegetables by High Performance Liquid Chromatography (HPLC)-Diode Array Detector (DAD)

The polyphenol amounts were analyzed by HPLC in combination with DAD as described in our previous report [25] with small modifications. Briefly, the extracted residues described above were dissolved in 0.5% trifluoroacetic acid containing methanol as amounts of 50 mg/mL, filtered through a 0.2 μm membrane filters (Millex-LG, Millipore, Billerica, MA, USA), and then analyzed by HPLC system, in which system was a LC-NetII/ADC system control program (JASCO, Tokyo, Japan) equipped with a ChromNAV chromatography data station, PU-2089 Plus pump, AS-2057 Plus autosampler, CO-20600 Plus column oven, and MD-2018 Plus DAD system to monitor between 200 nm and 600 nm as wavelengths. The column, Capcell Pak UG120 (3.0 mm i.d. × 150 mm, 3 μm, OSAKA SODA Co. Ltd., Osaka, Japan), was used at 40 °C. Linear gradient elution was performed with mobile phase A, comprising 50 mM sodium phosphate (pH 3.3) and 10% methanol and mobile phase B, 70% methanol, delivered at a flow rate of 0.4 mL/min as follows: initially 100% of A; for the next 15 min, 70%; for another 30 min, 65% A; for another 20 min, 60% A; for another 5 min, 50% A; and finally, 0% A for 20 min. The injection volume for the extract was 4 µL.

Additionally, the extracts were subjected to acidic hydrolysis in order to check the aglycone structures of flavonoids according to our previous reports with small modification [24]. Briefly, freeze-dried powders (50 mg) were placed in the test tube and mixed with 4 mL of 62.5% aqueous methanol containing 0.5 mg/mL of tert-butylhydroquinone and 1 mL of 2 N hydrochloric acid. The tubes were heated at 90 °C for 2 h, and then the samples were extracted with two volumes of ethyl acetate. The ethyl acetate fractions were collected, and dried with a CC-105 centrifugal concentrator (Tomy Seiko Co., Ltd., Tokyo, Japan). The residues were dissolved in 0.5% trifluoroacetic acid containing methanol, and then applied into the HPLC system described above.

### 4.6. Statistical Analysis

All the data were shown as mean ± standard error (SE). We performed Student’s *t*-test for comparison between two groups or one-way ANOVA and then post-hoc test Tukey-Kramer test for comparison among more than three groups as parametric analysis. To make comparisons within the groups, alpha value was set to 0.05.

## 5. Conclusions

Simultaneous consumption of vegetables containing high levels of quercetin and kaempferol was found to reduce the intake of fucoidan by approximately half while maintaining its antiallergic effect. This suggests that combinations of food factors have the potential to enhance physiological activity.

## Figures and Tables

**Table 1:** Flavonoids and phenolic acids in vegetable extracts.

	Flavonoids		Phenolic Acids	
	μmol/g Extracts		μmol/g Extracts	
Onion	quercetin-3,4’-diglucosides	4.81		–
	quercetin-4’-glucoside	5.30		
	quercetin-7-glucoside	0.20		
	other quercetin glucosides	1.18		
	quercetin	0.07		
Red bell pepper	quercetin-3-rhamnoside	0.77	cinnamic acids	0.32
	luteolin glucosides	1.99		
Welsh onion	kaempferol glycosides	3.15	cinnamic acids	3.36
Storage ginger		–		–
Radish		–	cinnamic acids	2.58

## Data Availability

Not applicable.

## Abbreviations

DAD	diode array detector
Gal9	galectin-9
HPLC	high performance liquid chromatography
PCA	passive cutaneous anaphylaxis
TNP	2,4,6-trinitrophenyl
